# DETECTION OF DEPRESSION USING THE UCLA THREE-ITEM LONELINESS SCALE IN OLDER CHINESE

**DOI:** 10.1093/geroni/igz038.1951

**Published:** 2019-11-08

**Authors:** Tianyin Liu, Gloria H Y Wong, Jennifer Y M Tang, Shiyu Lu, Dara Leung, Lesley Sze, Joyce Sing, Terry Lum

**Affiliations:** 1 The University of Hong Kong, Hong Kong, Hong Kong; 2 Sau Po Centre on Ageing, The University of Hong Kong, Pok Fu Lam, Hong Kong; 3 Department of Social Work and Social Administration, PokFuLam, Hong Kong

## Abstract

Loneliness is a significant and independent risk factor for later life depression. This study aimed to validate the Chinese version of the UCLA 3-item Loneliness Scale and to ascertain the cut-off point in detecting depression in community-dwelling older adults. The English version of the UCLA was translated into Chinese by six experienced social workers, clinical psychologists and researchers, and content validity was established by consultation and revision with 10 older adults. 1,919 older adults aged 60 years and over (average age = 76.3±8.0) were recruited from local NGOs, they were interviewed for demographic information, and assessed using the 3-item loneliness scale and the Patient Health Questionnaire (PHQ-9). Cronbach’s α of the Chinese loneliness scale was 0.87; the average score was 4.0 ± 3.0 out of 9, and it significantly correlated with living alone (r = 0.18, p < 0.001), unmarried (r = 0.12, p < 0.001), no emotional support (r = 0.14, p < 0.001), and depression (r = 0.41, p < 0.001). Using PHQ-9 cut-off score of 5 for mild and above depression, the area under the curve (AUC) was 0.73 ± 0.1 (p < .001, 95% CI 0.71-0.76) with 77% sensitivity and 61% specificity. We determined a cut-off point of 3 for loneliness using the Youden index, which revealed better sensitivity over alternative definitions of loneliness. A cut-off point of 3 in the Chinese UCLA 3-item loneliness scale can reliably identify possible depression in community-dwelling older Chinese.

